# Clinical factors and outcomes associated with immune non-response among virally suppressed adults with HIV from Africa and the United States

**DOI:** 10.1038/s41598-022-04866-z

**Published:** 2022-01-24

**Authors:** Adi Noiman, Allahna Esber, Xun Wang, Emmanuel Bahemana, Yakubu Adamu, Michael Iroezindu, Francis Kiweewa, Jonah Maswai, John Owuoth, Lucas Maganga, Anuradha Ganesan, Ryan C. Maves, Tahaniyat Lalani, Rhonda E. Colombo, Jason F. Okulicz, Christina Polyak, Trevor A. Crowell, Julie A. Ake, Brian K. Agan

**Affiliations:** 1 Infectious Disease Clinical Research Program, Department of Preventive Medicine and Biostatistics, School of Medicine, Uniformed Services University of the Health Sciences, Bethesda, MD USA; 2 Henry M. Jackson Foundation for the Advancement of Military Medicine, Bethesda, MD USA; 3 US Military HIV Research Program, Walter Reed Army Institute of Research, Silver Spring, MD USA; 4 Henry M. Jackson Foundation MRI, Mbeya, Tanzania; 5 U.S. Army Medical Research Directorate-Africa, Nairobi, Kenya; 6 Henry M. Jackson Foundation MRI, Abuja, Nigeria; 7 Makerere University Walter Reed Project, Kampala, Uganda; 8 Kenya Medical Research Institute, Nairobi, Kenya; 9 Henry M. Jackson Foundation MRI, Kericho, Kenya; 10 Henry M. Jackson Foundation MRI, Kisumu, Kenya; 11 National Institute of Medical Research-Mbeya Medical Research Centre, Mbeya, Tanzania; 12 Walter Reed National Military Medical Center, Bethesda, MD USA; 13 Naval Medical Center San Diego, San Diego, CA USA; 14 Naval Medical Center Portsmouth, Portsmouth, VA USA; 15 Madigan Army Medical Center, Joint Base Lewis-McChord, WA USA; 16 Brooke Army Medical Center, San Antonio, TX USA; 17 Division of Infectious Diseases, Department of Medicine, School of Medicine, Uniformed Services University of the Health Sciences, Bethesda, MD USA; 18 Infectious Disease Clinical Research Program, Henry M. Jackson Foundation for the Advancement of Military Medicine, Uniformed Services University of the Health Sciences, 11300 Rockville Pike, Suite 600, Rockville, MD 20852 USA

**Keywords:** HIV infections, Predictive markers

## Abstract

A significant minority of people living with HIV (PLWH) achieve viral suppression (VS) on antiretroviral therapy (ART) but do not regain healthy CD4 counts. Clinical factors affecting this immune non-response (INR) and its effect on incident serious non-AIDS events (SNAEs) have been challenging to understand due to confounders that are difficult to control in many study settings. The U.S. Military HIV Natural History Study (NHS) and African Cohort Study (AFRICOS). PLWH with sustained VS (< 400 copies/ml for at least two years) were evaluated for INR (CD4 < 350 cells/µl at the time of sustained VS). Logistic regression estimated adjusted odds ratios (aORs) and 95% confidence intervals (CIs) for factors associated with INR. Cox proportional hazards regression produced adjusted hazard ratios (aHRs) for factors associated with incident SNAE after sustained VS. INR prevalence was 10.8% and 25.8% in NHS and AFRICOS, respectively. Higher CD4 nadir was associated with decreased odds of INR (aOR = 0.34 [95% CI 0.29, 0.40] and aOR = 0.48 [95% CI 0.40, 0.57] per 100 cells/µl in NHS and AFRICOS, respectively). After adjustment, INR was associated with a 61% increase in relative risk of SNAE [95% CI 1.12, 2.33]. Probability of "SNAE-free" survival at 15 years since sustained VS was approximately 20% lower comparing those with and without INR; nearly equal to the differences observed by 15-year age groups. CD4 monitoring before and after VS is achieved can help identify PLWH at risk for INR. INR may be a useful clinical indicator of future risk for SNAEs.

## Introduction

Advances in antiretroviral therapy (ART) have led to substantial improvements in viral suppression and increased life expectancy for people living with HIV (PLWH) in resource-rich and resource-limited settings^1,2^. ART that suppresses peripheral viremia below the limit of detection on standard clinical assays is typically linked to CD4 reconstitution^3^ that leads to improved health outcomes^4,5^. Nevertheless, a sizeable proportion of PLWH who achieve viral suppression (VS) do not experience CD4 reconstitution after starting treatment, with findings ranging from approximately 20% in high income settings such as the Danish HIV Cohort Study^6^ to over 50% in lower income settings such as northwestern Tanzania^7^. The inability to regain healthy CD4 counts despite suppressive ART is known as immune non-response (INR).

The exact definition of INR differs by setting, both in terms of viral load criteria for VS and CD4 criteria for immune reconstitution. For instance, the World Health Organization (WHO) broadly defines VS as a viral load less than 1000 copies/ml in low-and middle-income countries (LMIC) while guidelines for high income countries indicate that a viral load of less than 50 copies/ml signifies VS^8^. The duration of low-level HIV RNA concentration indicating VS has also varied across past studies investigating INR^9–11^. Similarly, a recent systematic review found 14 unique INR definitions across 20 studies utilizing both absolute and relative measures of CD4^12^. Despite these methodological differences, INR measures are believed to be independent predictors of disease progression among PLWH on ART^13^. Studies with different definitions have also consistently demonstrated associations between INR and increased risk for immune malfunction, immune activation, apoptosis and immunosenescence, as well as AIDS- and non-AIDS-related morbidity and mortality^14–16^.

While INR is a concern for PLWH, the causal pathway to this outcome and from INR to future adverse health outcomes is not fully understood. Clinical factors affecting INR and the effect of INR on the development of serious non-AIDS events (SNAEs) have been challenging to isolate, partly due to confounding by age, co-infections associated with immune activation, access to healthcare and socioeconomic status^17^. Furthermore, it has been difficult to compare results across studies due to the variations in INR definitions described above. This study applied consistent outcome and exposure definitions to measure rates of and factors associated with INR in two methodologically similar but demographically diverse cohorts providing equal access to healthcare: The U.S. Military HIV Natural History Study (NHS) and the African Cohort Study (AFRICOS). This study also further addressed the clinical relevance of INR by looking at its relationship to the development of SNAEs in the NHS.

## Methods

### Study population and design

The NHS and AFRICOS are ongoing longitudinal cohort studies of PLWH that regularly collect data related to demographics, medical histories, medications, vaccinations and clinical laboratory results. NHS enrollment began in 1986 and includes active duty U.S. military personnel and beneficiaries from the Army, Navy/Marines and Air Force seeking care at five military medical centers who are identified through routine and mandatory Department of Defense (DoD) screening. Individuals interested in enlisting must test negative for HIV upon entry into the U.S. military and routine HIV testing of active duty members happens every 1–5 years. This allows for a well-defined seroconversion window for incident HIV infection. Those who test positive for HIV during service are referred to military medical centers and receive evaluation, ongoing care and an invitation to enroll as participants in the NHS. All healthcare, medications and travel are provided at no cost to active duty service members and retirees. Previous publications have described this cohort in further detail^18,19^. AFRICOS enrollment began in 2013 and now includes randomly selected participants from 12 clinics across five PEPFAR-supported programs in Kenya, Tanzania, Uganda and Nigeria who were identified as living with HIV or at risk for HIV from existing clinic patient lists or other HIV studies. To recruit participants at risk for HIV, adult partners of AFRICOS enrollees living with HIV and seronegative adults from the community or program counseling and testing activities were invited to enroll in the study. All AFRICOS participants received the standard of care associated with their PEPFAR-supported site. The details of this study, as well as the characteristics of each study site, have also been previously described^20,21^. The NHS has been approved centrally by the Uniformed Services University (USU) Institutional Review Board (IRB) and at each participating site. AFRICOS has been approved by the Walter Reed Army Institute Research (WRAIR) IRB and at each participating site. Participants from both studies provided written informed consent. This manuscript describes independent retrospective analyses of existing data in each cohort that were jointly approved by the USU and WRAIR IRBs in accordance with all the relevant guidelines and regulations.

### Eligibility criteria

This study included PLWH from the NHS and AFRICOS who initiated ART between January 1, 1996 and August 31, 2017. ART was defined as at least three full-dose antiretroviral agents from at least two classes, or three nucleoside reverse transcriptase inhibitors (NRTIs), including abacavir. Included participants had achieved “sustained VS,” defined as consecutive viral load measures of ≤ 400 copies/ml for at least two years since starting treatment. This viral load cutoff was selected to account for the heterogeneity of the assay detection limits used in both cohorts over the study period. To be included in this study, participants were also required to have a CD4 measure available at the time that sustained VS was achieved (± 90 days).

### Outcomes and exposures of interest

All outcome and exposure variables were obtained from each study’s electronic database. The primary outcome was INR, defined as an absolute CD4 measure of < 350 cells/µl at the time of sustained VS. Participants with CD4 ≥ 350 at this timepoint were considered to exhibit good immune response (GIR). Absolute CD4 was used to define INR in lieu of percent or absolute change in CD4 because increases in CD4 counts since treatment initiation in virologically suppressed PLWH have been shown to depend on CD4 counts at ART initiation^22^. In addition, CD4 is considered a reflection of immune system health and some evidence suggests there is greater prognostic value with respect to risk of HIV progression and mortality as compared to changes in CD4 since treatment initiation^23,24^.

Routine data collection in the NHS includes capture of SNAEs diagnosed in routine clinical care. Incident SNAE after achieving sustained VS was therefore evaluated as a secondary outcome among NHS participants only. Cancer, cardiovascular, genitourinary, respiratory, liver, gastrointestinal, and musculoskeletal events described in supplemental Table 1 were considered SNAEs based on review of the literature^25,26^ and consultation with study investigators. SNAE diagnoses were obtained through NHS participant interviews and review of medical records.

**Table 1:** Demographics and clinical characteristics of PLWH in the NHS and AFRICOS.

Characteristic	NHS				AFRICOS			
	Total (n = 1784)	INR (n = 193)	No INR (1591)	*p* value	Total (n = 984)	INR (n = 254)	No INR (n = 730)	*p* value
**Median age** (years)^a^ (IQR)	34.2 (28.6, 40.2)	37.1 (31.9,42.5)	33.9(28.3, 40.0)	< 0.001	37.2 (31.0, 44.2)	40.0 (34.2, 47.1)	36.3 (30.3,42.9)	< 0.001
**Females** (%)	6.8	6.7	6.9	0.95	57.0	38.2	63.5	< 0.001
**BMI** (kg/m^2^) (%)^a^				0.21				0.052
Underweight (≤ 18.5)	0.7	1.6	0.6		9.4	12.2	8.4	
Overweight (> 25)	58.1	52.3	58.8		28.0	23.2	29.6	
**History of smoking** (%)^a b^	19.7	22.3	19.4	0.44	2.0	4.3	2.7	0.21
**History of alcohol use** (%)^a b^	15.4	15.0	15.5	0.55	15.0	19.29	13.44	0.024
**Hypertension** (%)^c^	19.7	29.5	18.5	< 0.001	13.8	12.6	14.1	0.54
**Diabetes** (%)^c^	4.3	11.4	3.5	< 0.001	11.5	12.6	11.1	0.52
**Depression** (%)^c^	20	27.5	19.1	0.0062	17.9	18.1	17.8	0.92
**Chronic HBV** (%)^c^		5.8	2.6	< 0.001	4.0	3.4	4.5	0.49
**Chronic HCV** (%)^c^	4.2	6.7	3.9	0.15	1.3	1.7	1.1	0.51
**AIDS event** (%)^c^	11.3	35.2	8.4	< 0.001	16.5	20.9	15.1	0.033
**ART adherence < 95%** (%)^b^	26.6	33.3	25.8	0.027	10.1	10.2	10.0	0.92
**Median CD4 nadir** (cells/µl) (IQR)	302.0 (213.0, 400.0)	144.5 (54.0, 212.0)	318.5(239.5, 414.0)	< 0.001	190.0 (91.0, 279.0)	103.5 (50.0, 184.0)	219.5 (118.0, 315.0)	< 0.001
**Median viral load**^a^ (log_10_ copies/ml) (IQR)	4.5 (3.8, 5.0)	4.6 (3.8, 5.2)	4.5 (3.8, 4.9)	0.012	7.79 (3.04, 11.6)	5.99 (3.69, 11.9)	7.80 (5.0, 11.6)	0.70
**ART regimen at time of VS** (%)				0.078				0.67
NNRTI	39.9	27.5	41.4		79.3	78.0	79.8	
Protease Inhibitor (PI)	20.7	19.2	20.9		5.4	5.9	5.2	
Boosted PI	15.8	22.8	14.9		N/A	N/A	N/A	
Integrase Inhibitor	3.6	2.1	3.8		N/A	N/A	N/A	
Other	20.0	28.4	19.0		15.3	16.1	15.0	
**Median time to ART initiation** (years) (IQR)	1.7 (0.2, 6.0)	4.7 (3.8, 5.2)	1.6 (0.2, 5.4)	< 0.001	0.4 (0.1, 2.0)	0.2 (0.1, 0.8)	0.5 (0.1, 2.3)	< 0.001
**Median time from ART initiation to sustained VS** (years) (IQR)	2.5 (2.3, 4.1)	3.2 (2.3, 8.4)	2.5 (2.3, 3.8)	< 0.001	4.8 (2.4, 7.2)	4.3 (2.3, 6.5)	5.0 (2.5, 7.6)	0.005
**Race** (%)				0.40	N/A	N/A	N/A	N/A
Caucasian	43.0	47.2	42.6					
African American	41.0	39.4	41.2					
Other	16.0	13.5	16.0					
**Military rank** (%)^a^				< 0.001	N/A	N/A	N/A	N/A
Officer/Warrant	10.3	6.7	10.7					
Enlisted	63.6	54.4	64.7					
Dependent	26.1	38.9	24.5					
**SNAE** (%)^c^	8.7	16.6	7.8	< 0.001	N/A	N/A	N/A	N/A
**Study site** (%)		N/A	N/A	N/A				< 0.001
Uganda					23.6	22.4	24.0	
Kenya (SRV)					41.9	50.8	38.8	
Kenya (Kisumu)					18.3	11.0	20.9	
Tanzania					7.6	11.4	6.3	
Nigeria					8.5	4.3	10.0	
**≥ Secondary education** (%)^a^	N/A	N/A	N/A	N/A	36.9	24.8	27.6	0.37
**Employed** (%)^a^	N/A	N/A	N/A	N/A	45.8	42.5	46.9	0.23
**TB coinfection** (%)^c^	N/A	N/A	N/A	N/A		7.1	4.7	0.14

^a^At ART initiation.

^b^Definitions and ascertainment methods for these indicators are described in supplemental Table 2.

^c^Defined as a diagnosis occurring before sustained VS was achieved.

In this analysis, exposures at ART initiation in both cohorts included age (years), sex (male/female) history of smoking (yes/no), history of alcohol use (yes/no), history of depression (yes/no), body mass index (BMI) (kg/m^2^) and viral load (copies/ml). CD4 nadir (cells/µl), time from HIV diagnosis to ART initiation (years), time from ART initiation to sustained VS (years), ART regimen at time of VS (non-nucleoside reverse transcriptase inhibitor (NNRTI), protease inhibitor (PI), boosted PI, integrase inhibitor or other) and ART adherence were also considered exposures of interest. Any chronic hepatitis B (HBV)^27^, chronic hepatitis C (HCV)^28^, hypertension^29^, diabetes^30^ or AIDS event^31^ diagnoses (yes/no) before sustained VS were also included due to previously shown associations with inflammation in the literature. Ascertainment methods (supplemental Table 2) were similar in both studies, except for ART adherence and alcohol use. Adherence was reported as the average proportion of days covered between ART initiation and sustained VS using pharmacy data in the NHS and as the self-reported number of missed doses in the past month in AFRICOS. Alcohol use was self-reported in both cohorts but defined as “at-risk” drinking^32^ in the NHS and “any alcohol use” in AFRICOS. The NHS analysis also included race (Caucasian, African American or other) military rank (officer/warrant, enlisted or dependent), and SNAE before sustained VS while the AFRICOS analysis included study site (Uganda, Southern Rift Valley (Kenya), Kisumu (Kenya), Tanzania or Nigeria) baseline education (none or some primary, primary or some secondary, secondary and above), employment status (yes/no) and TB co-infection before sustained VS (yes/no).

### Statistical analysis

Independent but identical analyses for the INR outcome were conducted for each cohort. Chi-squared and Wilcox–Mann–Whitney tests were performed to look at differences by immune response status in categorical and continuous exposure variables, respectively. Logistic regression was utilized to identify associations between exposures of interest and odds of INR. Significant results from the univariate comparisons (*p* < 0.05) were included in a stepwise selection process to create the adjusted logistic regression models and estimate adjusted odds ratios (aOR) for each cohort, and factors hypothesized to influence immune response were also kept in the model. The final model included all the covariates found to be significantly associated with immune non-response in the independent analyses conducted for each cohort. The Hosmer–Lemeshow test was applied to the final models to assess goodness of fit. A sensitivity analysis was also performed by repeating the steps above while excluding participants with CD4 > 350 cells/µl at ART initiation. The goal of this analysis was to check for potential bias associated with the fact that participants with a higher CD4 when starting therapy may be more likely to maintain a high CD4.

In the NHS, Cox proportional-hazards modeling was used to estimate the effect of INR on the relative risk of incident SNAE after achieving sustained VS. Unadjusted and adjusted hazard ratios (HRs) were calculated for INR status and covariates found to significantly affect INR, as well as known risk factors for SNAEs. Individual HRs for known risk factors of SNAE were calculated separately by INR status to look for notable changes in effect size by immune response status and assess the need for an interaction term in the final model. Kaplan–Meier methods with censoring due to death or loss to follow-up were used to estimate differences in time from sustained VS to incident SNAE by immune response status, age at ART initiation and nadir CD4. NHS analyses were performed using SAS software, version 9.4 (Cary, NC) and AFRICOS analyses were performed with Stata statistical software, version 14.0 (College Station, TX).

## Results

### Description of the cohorts

Of the 3380 NHS participants eligible for this analysis, 564 (16.7%) had not achieved sustained VS by August 31, 2017 and 743 (21.9%) had less than two years of viral load measurements because they were either no longer eligible for DoD benefits (n = 244), lost to follow-up (n = 226), on ART for less than two years at the time of data collection (n = 160), deceased (n = 64) or transferred/withdrawn from the study (n = 49). Out of 1784 NHS participants with at least 2 years VS and an available CD4 measure who were eligible for this study, 193 (10.8%) met the definition for INR.

Of the 2625 AFRICOS participants eligible for this analysis, 313 (12.0%) had not achieved sustained VS by August 31, 2017 and 1316 (50.1%) had less than two years of viral load measurements because they were either on ART for less than two years (n = 1148), lost to follow-up (n = 115) or deceased. (n = 53). Out of 984 AFRICOS participants with at least two years VS and an available CD4 measure who were eligible for this study, 254 (25.8%) experienced INR.

Table 1 summarizes the demographic and clinical characteristics of each study population by immune response status. Compared to the NHS, the AFRICOS population was older (37.2 years vs. 34.2 years), with a larger proportion of females (57.0% vs. 6.8%) and a smaller proportion of smokers (2.0% vs. 19.7%) and overweight participants (28.0% vs. 58.1%). Median CD4 nadir was higher in the NHS (302 cells/µl vs. 190.0 cells/µl), and while the median time from HIV diagnosis to ART initiation was longer in the NHS than AFRICOS (1.7 years vs. 0.4 years), the median time from ART initiation to VS was shorter (2.5 years vs. 4.8 years). At the time of viral suppression, 79.3% and 5.4% of the AFRICOS population were on NNRTIs and PIs respectively, compared to 39.9% and 20.7% of the NHS population. In both cohorts, immune non-responders were significantly older, had a significantly lower CD4 nadir and a significantly longer time from ART initiation to VS, compared to those with GIR. Time from HIV diagnosis to ART initiation was also significantly different by immune response status in both cohorts, though it was longer for immune non-responders in the NHS and shorter in the AFRICOS.

### Odds of immune non-response

Table 2 reports the crude and adjusted odds ratios (aORs) for factors found to be significantly different by immune response status in each cohort. In both cohorts, CD4 nadir was significantly associated with INR in the adjusted models, such that an increase in CD4 was associated with decreased odds of INR (aOR = 0.34 [95% CI 0.29, 0.40] and aOR = 0.48 [95% CI 0.40, 0.57] in NHS and AFRICOS, respectively). Hypertension was also associated with INR in the NHS model, such that odds of INR increased by 71% for those with a hypertension diagnosis (*p* < 0.005). In the AFRICOS analysis, age at ART initiation and sex were also significantly associated with odds of INR in the adjusted model, such that odds of INR increased 5% for every year increase in age (*p* < 0.001) and decreased by 48% for women versus men (*p* = 0.001). Table 3 reports the adjusted multiple logistic regression models excluding participants with CD4 > 350 cells/µ at ART initiation. For both cohorts the models remained similar to the primary models, though instead of hypertension, diabetes became independently associated with INR among the restricted sensitivity analysis population of the NHS. The Hosmer–Lemeshow test for model fitness found the adjusted models in the NHS and AFRICOS to provide adequate fit (chi-square = 13.067; *p* = 0.12 and chi-square = 4.58; *p* = 0.80, respectively).

**Table 2:** Crude and adjusted multiple logistic regression results for factors associated with INR in the NHS and AFRICOS.

Characteristic	Crude OR^a^ (95% CI)	*p* value	Adjusted OR^b^ (95% CI)	*p* value
**Natural history study**				
History of hypertension^c^ (reference group = no)	1.84 (1.32, 2.57)	< 0.001	1.71 (1.17, 2.52)	0.006
CD4 nadir (cells/µl) (per 100 cells)	0.36 (0.31, 0.41)	< 0.001	0.34 (0.29, 0.40)	< 0.001
Age at ART initiation (years)	1.04 (1.02, 1.06)	< 0.001	1.01 (0.99, 1.03)	0.517
Sex (reference group = male)	0.98 (0.54, 1.78)	0.953	0.68 (0.35, 1.33)	0.257
**African cohort study**				
History of hypertension^c^ (reference group = no)	0.88 (0.57, 1.34)	0.542	0.59 (0.36, 0.95)	0.301
CD4 nadir (cells/µl) (per 100 cells)	0.50 (0.43,0.58)	< 0.001	0.48 (0.40, 0.57)	< 0.001
Age at ART initiation (years)	1.04 (1.03, 1.06)	< 0.001	1.05 (1.03. 1.06)	< 0.001
Sex (reference group = male)	0.35 (0.26, 0.48)	< 0.001	0.52 (0.37, 0.72)	0.001

^a^Crude ORs were calculated for all factors with significant differences by immune response status as per chi-squared and Wilcox–Mann–Whitney tests (*p* < 0.05).

^b^Stepwise selection was used to create the adjusted logistic regression model. Factors significant in the crude analyses that were not significant in the adjusted models included: Age at ART initiation (NHS); time from HIV + to ART initiation (both cohorts); time from ART initiation to sustained VS (both cohorts); history of depression (NHS); history of diabetes (NHS); history of chronic HBV (NHS); poor ART adherence (NHS); history of AIDS event (both cohorts) and history of SNAE before sustained (NHS).

^c^Defined as a diagnosis occurring before sustained VS was achieved.

**Table 3:** Adjusted multiple logistic regression results in NHS and AFRICOS, excluding participants with CD4 nadir > 350 cells/µl.

Characteristic	Adjusted OR (95% CI)	*p* value
**Natural history study**^a^		
**Age at ART initiation** (years)	1.00 (0.99, 1.03)	0.506
**Sex**		
Female	0.67 (0.34, 1.33)	0.254
Male	Reference	
**CD4 nadir** (cells/µl) (per 100 cells)	0.34 (0.28, 0.41)	< 0.001
**History of diabetes**^**c**^		
Yes	2.17 (1.11, 4.25)	0.024
No	Reference	
**African Cohort Study**^b^		
**Age at ART initiation** (years)	1.05 (1.03, 1.07)	< 0.001
**Sex**		< 0.001
Female	0.49 (0.35, 0.71)	
Male	Reference	
**CD4 nadir** (cells/µl) (per 100 cells)	0.46 (0.37, 0.56)	< 0.001
**History of diabetes**^**c**^		
Yes	0.87 (0.52, 1.46)	0.606
No	Reference	

^a^842 participants in NHS with baseline CD4 > 350 cells/µl excluded.

^b^210 participants in AFRICOS with baseline CD4 > 350 cells/µl excluded.

^c^Defined as a diagnosis occurring before sustained VS was achieved.

### Relative risk of incident serious non-AIDS event

Table 4 reports the Cox proportional-hazards modeling results for hazard of incident SNAE in the NHS, with INR as the primary exposure of interest. In addition to INR, the final adjusted model included age, BMI and history of smoking at ART initiation, as well as history of hypertension, depression, diabetes and “at-risk” drinking. Crude hazards ratios stratified by immune response status did not provide enough evidence for effect modification and interaction terms were therefore not included in the model.

**Table 4:** Crude and adjusted Cox proportional hazards model results for factors associated with incident SNAE in the NHS.

Characteristic	Crude HR^a^ (95% CI)	*p* value	Adjusted HR^b^ (95% CI)	*p* value
**Age at ART initiation** (years)	1.07 (1.05, 1.08)	< 0.001	1.07 (1.05, 1.08)	< 0.001
**History of smoking at ART initiation**		0.054		0.14
Yes	1.70 (0.99, 2.91)		1.51 (0.88, 2.61)	
No	Reference		Reference	
**BMI at ART initiation** (kg/m^2^)		0.43		0.24
Overweight (> 25)	0.99 (0.74, 1.31)		0.93 (0.7, 1.25)	
Underweight (≤ 18.5)	0.45 (0.06, 3.27)		0.31 (0.04, 2.21)	
Normal (18.5–24.9)	Reference		Reference	
**History of “at risk drinking” at ART initiation**		0.011		0.114
Yes	0.30 (0.12, 0.76)		0.31 (0.12, 0.79)	
No	Reference		Reference	
**History of hypertension** ^c^		0.47		0.11
Yes	1.14 (0.8, 1.63)		0.72 (0.48, 1.08)	
No	Reference		Reference	
**History of depression** ^c^		0.015		0.073
Yes	1.49 (1.08. 2.05)		1.34 (0.97, 1.85)	
No	Reference		Reference	
**History of diabetes** ^c^		< 0.001		0.47
Yes	2.33 (1.44, 3.79)		1.22 (0.72, 2.07)	
No	Reference		Reference	
**Immune non-response**		< 0.001		0.010
Yes	1.89 (1.32, 2.70)		1.61 (1.12, 2.33)	
No	Reference		Reference	

^a^Crude HRs were calculated for factors with significant differences by SNAE status as per chi-squared and Wilcox–Mann–Whitney tests (*p* < 0.05).

^b^Factors found to be significant in the crude analyses and known risk factors for SNAE (smoking, BMI, hypertension, depression, diabetes and alcohol use) (*p* < 0.05) were included in the adjusted model.

^c^Defined as a diagnosis occurring before sustained VS was achieved.

After adjustment, age and INR remained statistically significant, such that a one-year increase in age was associated with a 7% increase in the relative risk of incident SNAE (*p* < 0.001), and INR was associated with a 61% increased relative risk of SNAE compared to GIR (*p* = 0.0098).

### Time to incident serious non-AIDS event

**Figure 1:**
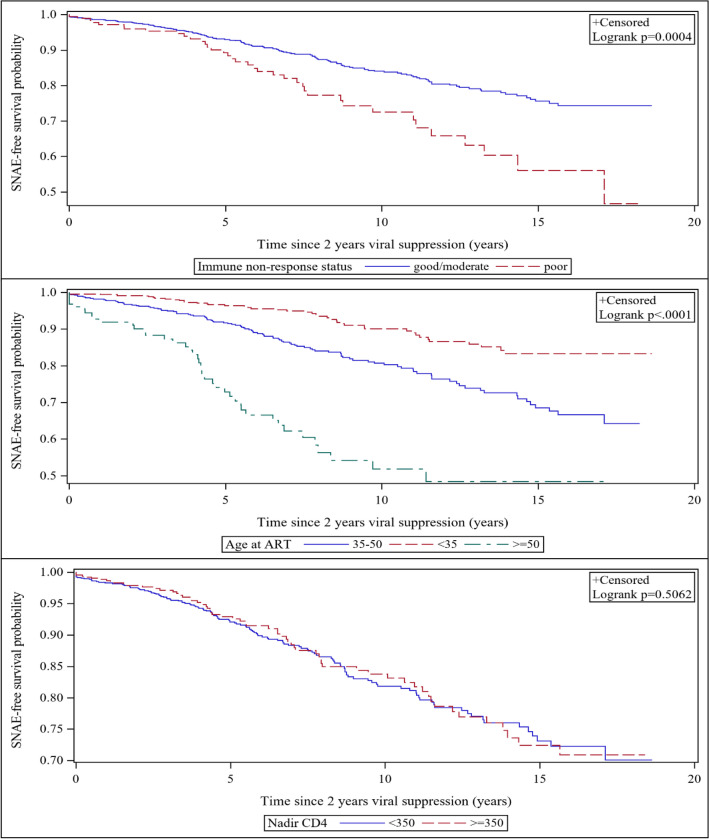
Time-to-incident SNAE. The first panel illustrates the difference in time-to-SNAE by INR status, with the blue representing CD4 ≥ 350 cells/µl and red representing CD4 < 350 cells/µl at viral suppression. The second panel illustrates the difference by age, with red representing < 35, blue representing 35–50 and green representing ≥ 50 years at ART initiation. The third panel represents the difference by nadir CD4, with blue representing < 350 cells/µl and red representing ≥ 350 cells/µl. The probability of “SNAE-free” survival 15 years after viral suppression was approximately 20% lower comparing those with and without INR; nearly equal to the difference observed by 15-year age groups.

The Kaplan–Meier curves in Fig. 1 depict the time to incident SNAE after sustained VS as survival probabilities stratified by immune response status, age at ART initiation and nadir CD4. Median time to incident SNAE was approximately six months longer for NHS participants with GIR compared to INR (13.74 years vs. 13.19 years, *p* < 0.001). The probability of "SNAE-free" survival at 15 years since sustained VS was approximately 20% lower comparing those with and without INR (*p* < 0.001); nearly equal to the differences observed by 15-year age groups. Median time to new SNAE was not significantly different by CD4 nadir (14.56 years for CD4 < 350 vs. 13.68 years for CD4 ≥ 350, respectively; *p* = 0.51).

## Discussion

These analyses of data from two distinct cohorts with limited confounding due to age, co-infections associated with immune activation, access to healthcare and socioeconomic status evaluated INR in PLWH from the NHS and AFRICOS who achieved sustained VS and assessed risk of and time to incident SNAE in the NHS cohort. INR was more common among AFRICOS participants than the U.S-based NHS. Despite cohort differences, higher CD4 nadir was protective against INR in both cohorts. INR was independently associated with an increased risk of incident SNAE in the NHS.

Our finding that lower CD4 nadir was associated with an increased odds of INR is consistent with other studies^16,33^, including an analysis by the Strategic Timing of Antiretroviral Treatment (START) trial that found lower initial CD4 counts to be associated with poor immune reconstitution, despite defining INR as an increase of < 50 CD4 cells/µl and limiting the analysis to participants who initiated treatment with a CD4 > 500 cells/µl^34^. These similar results despite different INR definitions highlight the importance of early HIV testing and treatment and continued CD4 monitoring before and after VS is achieved. A recent review by the WHO reported a shift away from using CD4 counts to determine treatment initiation or assess treatment efficacy^35^. However, our findings suggest that CD4 monitoring may play an important role in predicting INR, and that continued CD4 testing among those who successfully suppress HIV is important for detecting INR and potentially predicting future adverse health outcomes. The same review found that underutilization of CD4 instruments persists globally due to non-installation, limited capacity for instrument maintenance and lack of reagents for point-of-care CD4 testing, with only 13.7% of existing CD4 capacity being utilized across 50 countries reporting data in 2013. Given the high prevalence of INR, especially in LMIC such as the AFRICOS study sites, it is crucial to improve utilization of CD4 measurement tools in these settings by addressing these well-documented barriers to their use.

In the U.S.-based NHS cohort, a finding that warrants further investigation is the association between hypertension and INR. Although this relationship was not observed in the AFRICOS, new research suggests that the etiology of hypertension may be related to activated immune cells entering target organs in the vasculature and kidney and releasing mediators such as reactive oxygen species, metalloproteinases, cytokines and antibodies damage target organs^36^. Since some evidence indicates that INR may also be characterized by systemic immune activation^37^, it is possible that a hypertension diagnosis in this case is a marker of the immune activation shown to be associated with INR. Further research is needed to better understand the cellular and molecular pathways involved in immune reconstitution, and studies to identify these mechanisms are underway.

Our study also found INR to be strongly associated with an increased risk of incident SNAE after achieving sustained VS, even while controlling for known risk factors such as age, smoking status, BMI, hypertension, depression, diabetes and alcohol consumption. An analysis from the Italian MASTER Database Cohort also found risk of SNAE to increase among PLWH unable to achieve immune reconstitution, but this cohort was significantly older (median age = 42.1) and restricted to those with a CD4 < 200 cells/µl at treatment initiation^38^. Our finding suggests that INR may be independently linked to SNAE, even among a younger and healthier study population of PLWH with equal access to healthcare. It is possible that the INR phenotype represents a predisposition or biomarker for underlying immune activation as described above and is in fact a mediator in the causal pathway between immune system dysfunction and SNAE. Further research is needed to examine the effects of inflammation on incident SNAE. For instance, follow-up analyses could assess the level of chronic and persistent inflammation in PLWH who exhibit INR. It could also be beneficial to look at changes in inflammatory markers over time and measure the effects of drugs with anti-inflammatory properties such as angiotensin-converting enzyme (ACE) inhibitors and statins. Similarly, neurological and mental health outcomes were excluded in the study definition of SNAE but complementary analyses may be warranted to look specifically and extensively at the association between INR and diseases related to neurological and mental health. Nevertheless, INR may be a predictor of future SNAE that could be determined quickly and easily in clinical settings to identify higher-risk patients. INR measures (i.e. CD4) may also be more readily available in resource-limited settings where laboratory methods for detecting immunosenescence, inflammation or altered cellular metabolic pathways, such as transcriptional profiling and polychromatic flow cytometry, are less common.

It has also been shown that caring for an aging population of PLWH who are at increased risk for SNAEs is associated with increased healthcare costs^39^. Our finding that INR is independently associated with an increased risk for SNAEs suggests that in addition to interventions aimed at reducing the effect of known risk factors such as smoking, drinking or obesity, more work must be done to identify interventions that can successfully prevent or reverse the effects of INR. Such interventions may in turn decrease the incidence of SNAEs, improve health outcomes for aging PLWH and help to alleviate associated economic strains on the healthcare system.

Since SNAE diagnoses are known to be age-associated comorbidities, it is not surprising that the burden of these comorbidities among PLWH has increased as survival has improved. However, existing evidence has not definitively shown if HIV to causes accelerated or accentuated aging^40^. For instance, the Veterans Aging Cohort Study (VACS) found PLWH had a higher risk of age-associated comorbidities, but that events happened at similar ages as those without HIV^41^, while a case control study in Italy found the prevalence of multiple comorbidities in PLWH to equal the prevalence observed in HIV negative controls who were 10–15 years older^42^. Although our study did not include an HIV-uninfected population, the results from our survival analysis may be useful for this discussion. As expected, we found that NHS participants ≥ 50 years old had the shortest time to incident SNAE after achieving sustained VS. More interestingly, immune response status played an equally and independently significant role in progression to a SNAE, such that the difference in time-to-event comparing INR to GIR was approximately the same magnitude and direction as the difference observed by 15-year increases in age. This suggests that INR should be considered in future work modeling the relationship between HIV and aging.

There are some limitations to this study. First, SNAE data were not available from AFRICOS at the time of this analysis so the effect of INR on development of these comorbidities could not be ascertained in the lower-resource setting. Second, SNAEs were not stratified by disease category (e.g. cardiovascular, cancer, etc.) due to sample size limitations. To increase the power of this analysis, an aggregate measure of any incident SNAE was used, but it is possible that the relationship between INR and SNAE may differ by disease category. Third, while some relevant coinfections such as chronic HBV and HCV were included in the analysis, information about other infections that may affect HIV virulence, such as human papillomavirus (HPV), were not available to include in this analysis. Similarly, additional data about factors that are known to be associated with inflammation, such as utilization of vitamin D, were not available for this analysis and were therefore excluded. Fourth, this study did not include markers of inflammation such as interleukin-6 (IL-6) and tumor necrosis factor alpha (TNF alpha) to measure levels of inflammation. Fifth, it is feasible that neurocognitive and mental health diagnoses may be associated with INR since both outcomes may be associated with inflammation^43^ but these diagnoses were excluded from the study due to considerable differences in how these diagnoses were defined and ascertained in the two cohorts. Sixth, a recent AFRICOS analysis reports that time to ART initiation varies by PEPFAR country^44^, which may lead to differences in odds of INR by study site that cannot be detected with the aggregate measure used in this analysis. However, the same AFRICOS analysis also showed that the differences in time to ART initiation by country decreased significantly by 2013, so the effect on INR should also become less significant over time. Finally, INR was treated as a cross-sectional measure, but there may be some benefit to treating INR as time-varying in order to assess the effect of changing INR status on the development or severity of SNAEs.

## Conclusions

Our study highlighted that lower CD4 nadir was significantly associated with INR, regardless of study setting or demographics, and that INR was significantly associated with risk of SNAE. Given the changing policies surrounding CD4 testing and the increased life expectancy of PLWH, special attention should be paid to the benefits of identifying and monitoring poor immune responders and testing interventions to prevent or reduce the effects of INR.

## Supplementary Information

Supplementary Tables.
