# Supplementary material for: Clinical factors and outcomes associated with immune non-response among virally suppressed adults with HIV from Africa and the United States
Source: Sci Rep. 2022 Jan 24;12:1196. doi: 10.1038/s41598-022-04866-z (PMC8786968; doi:10.1038/s41598-022-04866-z)
Supplement: Supplementary file 1 — Supplementary Tables. [file 41598_2022_4866_MOESM1_ESM.docx]

**Complete manuscript title:** Clinical factors and outcomes associated with immune non-response among virally suppressed adults with HIV from Africa and the United States

**Authors:** Adi Noiman PhD^1,2*^; Allahna Esber, PhD^2,3^; Xun Wang, MS^1,2^; Emmanuel Bahemana, MBBS^3,4^; Yakubu Adamu, MD, FMCPH^3,5,6^; Michael Iroezindu, MBBS, MPH^3,5,6^; Francis Kiweewa, MBBS^7^; Jonah Maswai, MBBS^3,8,9^; John Owuoth, MBBS^3,8,10^; Lucas Maganga, MD, MPH;^11^ Anuradha Ganesan, MBBS, MPH^1,2,12^; Ryan C. Maves, MD^13^; Tahaniyat Lalani, MBBS, MHS^1,2,14^; Rhonda E. Colombo, MD, MHS;^1,2,15^; Jason F. Okulicz, ^MD1,16^; Christina Polyak, MD, MPH^2,3^; Trevor A. Crowell, MD, PhD^3,17^; Julie A. Ake, MD, Msc^3,17^; Brian K. Agan, MD^1,2^

^1^ Infectious Disease Clinical Research Program, Department of Preventive Medicine and Biostatistics, School of Medicine, Uniformed Services University of the Health Sciences, Bethesda, MD

^2^ Henry M. Jackson Foundation for the Advancement of Military Medicine, Bethesda, MD

^3^ US Military HIV Research Program, Walter Reed Army Institute of Research, Silver Spring, MD

^4^ Henry M. Jackson Foundation MRI, Mbeya, Tanzania

^5^ U.S. Army Medical Research Directorate-Africa, Nairobi, Kenya

^6^ Henry M. Jackson Foundation MRI, Abuja, Nigeria

^7^ Makerere University Walter Reed Project, Kampala, Uganda

^8^ Kenya Medical Research Institute, Nairobi, Kenya

^9^ Henry M. Jackson Foundation MRI, Kericho, Kenya

^10^ Henry M. Jackson Foundation MRI, Kisumu, Kenya

^11^ National Institute of Medical Research-Mbeya Medical Research Centre, Mbeya, Tanzania

^12^ Walter Reed National Military Medical Center, Bethesda, MD

^13^ Naval Medical Center San Diego, San Diego, CA

^14^ Naval Medical Center Portsmouth, Portsmouth, VA

^15^ Madigan Army Medical Center, Joint Base Lewis-McChord, Washington

^16^ Brooke Army Medical Center, San Antonio, TX

^17^ Division of Infectious Diseases, Department of Medicine, School of Medicine, Uniformed Services University of the Health Sciences, Bethesda, MD

***Corresponding author:**

Adi Noiman, Ph.D.

Infectious Disease Clinical Research Program

Henry M. Jackson Foundation for the Advancement of Military Medicine

Uniformed Services University of the Health Sciences

11300 Rockville Pike, Suite 600

Rockville, MD 20852

Phone: (301) 881-2339

Email: [anoiman@idcrp.org](mailto:anoiman@idcrp.org)

**Supplemental table 1.** Serious non-AIDS events among those who achieved sustained viral suppression in the NHS cohort

| **SNAE diagnosis** | **Total (%)**  (n=1784) | **Good immune responders (%)** (n=1591) | **Poor immune responders (%)**  (n=193) |
| --- | --- | --- | --- |
| **CANCER** | | | |
| Anal | 0 (0.0) | 0 (0.0) | 0 (0.0) |
| Leukemia | 0 (0.0) | 0 (0.0) | 0 (0.0) |
| Colon | 1 (0.1) | 1 (0.1) | 0 (0.0) |
| Lung | 1 (0.1) | 1 (0.1) | 0 (0.0) |
| Multiple myeloma | 1 (0.1) | 0 (0.0) | 1 (0.5) |
| Breast | 5 (0.3) | 1 (0.1) | 4 (2.1) |
| Melanoma | 6 (0.3) | 5 (0.3) | 1 (0.5) |
| Hodgkin’s Lymphoma | 7 (0.4) | 6 (0.4) | 1 (0.5) |
| Prostate | 16 (0.9) | 15 (0.9) | 1 (0.5) |
| Other | 27 (1.5) | 23 (1.4) | 4 (2.1) |
| Total | 65 (3.6) | 53 (3.3) | 12 (6.2) |
|  |  |  |  |
| **CARDIOVASCULAR DISEASE** | | | |
| Pericardial effusion | 0 (0.0) | 0 (0.0) | 0 (0.0) |
| Myocarditis | 1 (0.1) | 1 (0.1) | 0 (0.0) |
| Congestive heart failure | 5 (0.3) | 4 (0.3) | 1 (0.5) |
| Pericarditis | 6 (0.3) | 6 (0.4) | 0 (0.0) |
| Peripheral artery disease | 7 (0.4) | 6 (0.4) | 1 (0.5) |
| Cerebrovascular disease | 10 (0.6) | 5 (0.3) | 5 (2.6) |
| Cardiomyopathy | 11 (0.6) | 9 (0.6) | 2 (1.0) |
| Myocardial infarction | 15 (0.8) | 13 (0.8) | 2 (1.0) |
| Deep vein thrombosis | 19 (1.1) | 15 (0.9) | 4 (2.1) |
| Coronary artery disease without myocardial infarction | 49 (2.7) | 38 (2.4) | 11 (5.7) |
| Total | 123 (6.9) | 97 (6.1) | 26 (13.5) |
|  |  |  |  |
| **GENITOURINARY DISEASE** | | | |
| Acute renal failure | 47 (2.6) | 37 (2.3) | 10 (5.2) |
|  |  |  |  |
| **RESPIRATORY DISEASE** | | | |
| Chronic obstructive pulmonary disease | 17 (1.0) | 12 (0.7) | 5 (2.6) |
| Pulmonary embolism | 4 (0.2) | 4 (0.3) | 0 (0.0) |
| Total | 21 (1.2) | 16 (1.0) | 5 (2.6) |
|  |  |  |  |
| **GASTROINTESTINAL AND LIVER DISEASE** | | | |
| Pancreatitis | 33 (1.9) | 27 (1.7) | 6 (3.1) |
| Cirrhosis | 13 (0.7) | 8 (0.5) | 5 (2.6) |
| Total | 46 (2.6) | 35 (2.2) | 11 (5.7) |
|  |  |  |  |
| **MUSCULOSKELETAL DISEASE** | | | |
| Avascular necrosis | 14 (0.8) | 12 (0.8) | 2 (1.0) |

**Supplemental table 2.** Ascertainment methods for exposures of interest in the INR analysis

| **Exposure** | **NHS ascertainment method and definition** | **AFRICOS ascertainment method and definition** |
| --- | --- | --- |
| History of smoking | **Method:** Self-report  **Definition:** Answering “yes” to the question: “have you ever smoked cigarettes?” | **Method:** Self-report  **Definition:** Answering “yes” to the question: “do you smoke cigarettes?” |
| BMI | **Method:** Medical record  **Definition***:* Ratio of weight to height in kg/m^2:^ Underweight: <18.5  Normal weight: 18.5-24.9  Overweight: ≥25 | **Method:** Medical record  **Definition:** Ratio of weight to height in kg/m^2:^ Underweight: <18.5  Normal weight: 18.5-24.9  Overweight: ≥25 |
| History of hypertension | **Method:** Clinical measure  **Definition:** NHS diagnosis code based on a systolic blood pressure >139 or diastolic blood pressure >89 or on hypertension  medications | **Method:** Clinical measure  **Definition:** Systolic blood pressure >139 or diastolic blood pressure >89 or on hypertension  medications |
| History of depression | **Method:** Medical record  **Definition:** Provider diagnosis of depression | **Method:** Self-report  **Definition:** CES-D^a^ Score ≥ 16 |
| History of diabetes | **Method:** Medical record  **Definition:** NHS diagnosis code based on fasting glucose >99 mg/dL, non-fasting glucose >199 mg/dL or receipt of hypoglycemic medications | **Method:** Clinical measure  **Definition:** Fasting glucose >99 mg/dL, non-fasting glucose >199 mg/dL  or receipt of hypoglycemic medications. |
| History of chronic HBV | **Method:** Clinical measure  **Definition:** ≥2 reactive hepatitis B surface antigen tests ≥ 6 months apart | **Method:** Clinical measure  **Definition:** ≥ 1 reactive hepatitis B surface antigen test |
| History of chronic HCV | **Method:** Clinical measure  **Definition:** ≥ 1 reactive anti-hepatitis C virus antibody test | **Method:** Clinical measure  **Definition:** ≥ 1 reactive anti-hepatitis C virus antibody test |
| History of AIDS event | **Method:** Medical record  **Definition:** ≥1 NHS diagnosis code for AIDS-defining condition or CD4 <200 cells/mm^3^, as per CDC diagnostic criteria ^b^ | **Method:** Medical record  **Definition:** WHO stage 3 or stage 4 event or CD4<200 cells/mm^3^, as per WHO diagnostic criteria.^c^ |
| ART adherence < 95% | **Method:** Pharmacy measure  **Definition:** Proportion of days covered between ART initiation and sustained VS <95% | **Method:** Self report  **Definition:** Proportion of missed doses in the past month > 95% |
| History of alcohol use | **Method:** Self-report  **Definition:** “At-risk drinking” defined as >4 drinks/day or >14 drinks/week for men and >3 drinks/day or >7 drinks/week for women, as per NIAAA criteria^d^ | **Method:** Self-report  **Definition:** Answering “yes” to the question: “do you currently use alcohol?” |
| History of TB | **Method:** Medical record  **Definition:** ≥1 NHS diagnosis code for TB before sustained VS was achieved. | **Method:** Clinical measure  **Definition:** ≥1 positive TB Xpert^e^ or mycobacterial smear or culture result |
| History of SNAE | **Method:** Medical record  **Definition:** *≥* 1 NHS diagnosis code representing one of the SNAEs described in supplemental table 1 before sustained VS was achieved | **Method**: N/A  **Definition:** N/A |

^a^ <https://cesd-r.com/about-cesdr/>

^b^ <https://www.cdc.gov/mmwr/preview/mmwrhtml/rr6303a1.htm?s_cid=rr6303a1_e>

^c^<https://www.who.int/hiv/pub/guidelines/HIVstaging150307.pdf>

^d^ <https://www.niaaa.nih.gov/alcohol-health/overview-alcohol-consumption/moderate-binge-drinking>

^e^ <https://www.cdc.gov/tb/publications/factsheets/pdf/xpertmtb-rifassayfactsheet_final.pdf>
